# Clinical outcomes for grades III–V acromioclavicular dislocations favor double-button fixation compared to clavicle hook plate fixation: a systematic review and meta-analysis

**DOI:** 10.1007/s00590-023-03492-3

**Published:** 2023-02-25

**Authors:** Erik Hohmann, Kevin Tetsworth

**Affiliations:** 1 Medical School, Faculty of Health Sciences, University of Pretoria, Pretoria, South Africa; 2 Department of Orthopaedic Surgery and Sports Medicine, Burjeel Hospital for Advanced Surgery, Dubai, UAE; 3 Department of Orthopaedic Surgery, Royal Brisbane Hospital, Herston, Australia; 4 Department of Surgery, School of Medicine, University of Queensland, Brisbane, Australia; 5 Limb Reconstruction Center, Macquarie University Hospital, Macquarie Park, Australia; 6 Orthopaedic Research Centre of Australia, Brisbane, Australia

**Keywords:** Acromioclavicular joint, Acute dislocation, Clavicle hook plate, Double-button repair, Clinical outcomes, Systematic review, Meta-analysis

## Abstract

**Introduction:**

The purpose of this study was to perform a systematic review and meta-analysis of both randomized controlled and observational studies comparing double-button suture fixation to hook plate fixation for types III–IV acromioclavicular joint dislocation.

**Methods:**

Systematic review of Medline, Embase, Scopus, and Google Scholar, including all levels 1–3 studies from 2000 to 2022. Clinical outcome scores, range of motion, and complications were included. Risk of bias was assessed using the Cochrane Collaboration’s ROB2 tool and ROBINs-I tool. MINORS and modified Coleman Methodology Score (CMS) were used to assess within study quality. The GRADE system was used to assess the overall quality of the body of evidence. Heterogeneity was assessed using *χ*^2^ and *I*^2^ statistics.

**Results:**

Fifteen studies were included. Three of the four included LOE II and eleven of the LOE III studies had a high risk of bias. Study quality was considered poor and fair for 67% by MINORS criteria and 93% for CMS criteria. The pooled estimate (SMD 0.662) for all clinical outcomes was statistically significant and in favor of button repair (*p* = 0.0001). The pooled estimate (SMD 0.662) for all VAS pain scores was statistically significant, again in favor of button repair (*p* = 0.001).

**Conclusions:**

The results of this meta-analysis demonstrated significantly better outcomes of button repair for acute ACJ dislocations when compared to clavicle hook plate. Button repair is also associated with a 2.2 times lower risk for complications. However, risk of bias is high, and study quality within and between studies was low. These results, therefore, must be viewed with caution.

**Level of evidence:**

Level III; systematic review and meta-analysis.

## Introduction

Acromioclavicular joint (ACJ) dislocations are common injuries, particularly in athletes involved in contact sports and victims of road traffic accidents [1, 2]. The mechanism is usually a direct fall onto the shoulder with the arm in adduction [1, 2]. The ACJ is stabilized by the acromioclavicular (AC) ligament and the coracoclavicular (CC) ligaments [3]. Disruption of the CC ligaments typically results in superior and horizontal displacement of the clavicle [4].

The amount of displacement has been classified by Rockwood [5]. Type I injuries represent a sprain of the AC ligament complex; type II injuries represent rupture of the ACL ligaments with no marked elevation of the clavicle [6]. These injuries should be treated non-operatively [2, 5, 6]. For types III–VI, both the AC ligament and CC ligaments are completely ruptured, and surgical treatment is generally recommended [2, 6].

Multiple surgical techniques have been described and include temporary coracoclavicular screw fixation, coracoacromial ligament transfer, CC ligament reconstruction, acromioclavicular stabilization with double-buttons, or hook plate fixation [2]. Arthroscopically assisted acromioclavicular joint stabilization using double-button suture devices via transclavicular-transcoracoid tunnels and hook plate fixation are contemporary techniques, and both can be considered standard techniques for acute injuries [6]. The suture button is a suspensory fixation technique that can be performed either open or arthroscopic assisted, augmenting the CC ligament complex with high-strength sutures [7]. Advantages of this single-stage technique are that associated glenohumeral pathologies can be treated concomitantly, and are reportedly well-accepted by patients. However, it is technically challenging and is associated with a 20–25% prevalence of implant irritation and iatrogenic clavicle and coracoid fractures [6]. The hook plate utilizes a special modified plate which is placed beneath the inferior surface of the acromion and fixed with screws on the superior aspect of the clavicle [8]. The advantages of this technique include early functional rehabilitation and low technical requirements, but it has been associated with acromial osteolysis, acromial fractures, and subacromial impingement pain [6].

Currently, there is no consensus as to which surgical technique is preferred. A recent meta-analysis has included only four studies which were published between 2014 and 2018, and concluded that both techniques provide good clinical outcomes but note double-button suture fixation resulted in less postoperative pain [9].

The purpose of this study was, therefore, to perform an updated systematic review and meta-analysis of both randomized controlled and observational studies comparing double-button suture fixation to hook plate fixation for types III–IV acromioclavicular joint dislocation.

## Methods

The updated guidelines described in the Cochrane Handbook [10] and the Preferred Reporting Items for Systematic Reviews and Meta-Analysis (PRISMA) guidelines [11] were used to conduct this study.

### Eligibility criteria

A comprehensive literature search for all studies comparing suture button to hook plate fixation for types III–VI acromioclavicular dislocations was performed on studies that fulfilled the inclusion and exclusion criteria. All levels I–III evidence studies were considered if they were published between January 2000 and August 2022. Level III studies were included to increase the sample size and increase the generalizability of the pooled results [12]. Several authors have demonstrated that the inclusion of LOE III studies does not cause any differences in the risk estimate of treatment effects of an intervention derived from randomized controlled trials, observational studies, or a combination of LOE I–III studies [12–14]. If studies compared various surgical interventions, or included a non-operative study arm, the data of interest were extracted from these studies and included in the analysis. Other inclusion criteria were defined as follows: minimum follow-up of 12 months and inclusion of at least one functional validated outcome scores (such as Constant, ASES, DASH, VAS, OSS, UCLA, or SANE). Level IV case series, abstracts, conference proceedings, and articles from non-peer-reviewed journals or unrefereed pre-publications were excluded. Although omission of gray data sources could have resulted in publication bias, it was considered that these publications would have been ineligible for inclusion.

### Literature search

All publications in English and German listed in the databases of Medline, Embase, Scopus, and Google Scholar were identified and checked against the eligibility criteria. The following terms and Boolean operators were utilized: “acromioclavicular” AND/OR “dislocation” AND/OR “injury” AND/OR “Rockwood”; AND/OR “suture button” AND/OR “TightRope” AND/OR “hook plate” AND/OR “operative treatment”; AND/OR “clavicle hook plate”; AND/OR “acromioclavicular joint.” For the Medline search, the following MeSH term “acromioclavicular joint” was used in addition to the above search strategy. In addition, a manual search of the included studies and published reviews was conducted for other potentially eligible studies. Two reviewers conducted independent title and abstract screening. Disagreements between reviewers were resolved by consensus, and if no consensus was reached, they were carried forward to the full-text review.

### Data extraction and quality assessment

An electronic data extraction form [Excel spreadsheet] was used to obtain the following data from each article: level of evidence, country, age, gender, length of follow-up, sample size, clinical outcome scores, and complications. Risk of bias was assessed using the Cochrane Collaboration’s Risk of Bias Tool [10] or LOE I and II studies. For LOE III studies, the ROBINS-I tool was used [15]. The GRADE system was used by two reviewers to assess the quality of the body of evidence for each outcome measure [10]. The recommendations from the Cochrane Handbook were followed, and an initial level of certainty assigned. Studies were downgraded if there was a high risk of bias, inconsistency and imprecision of the results, and indirectness of evidence. Studies were upgraded if there were large treatment effects, a dose–response, or reasons to oppose plausible residual bias and confounding effects. In addition to GRADE, the Methodological Index for Non-Randomized Studies (MINORS) and modified Coleman Methodology Score were used as a second validated instrument to assess the quality of the included surgical studies [16]. The MINORS score was categorized as per Ekhtiari et al. [17] into the following categories: < 6 very low quality, < 10 poor quality, < 14 fair quality, and > 16 good quality. Any disagreement between reviewers was resolved by consensus and/or by arbitration between the two senior authors.

### Statistical analysis

Interobserver differences for study eligibility and risk of bias were measured using Cohen’s kappa coefficient. Heterogeneity of the data was assessed using *χ*^2^ and *I*^2^ statistics. Outcomes were pooled using a random effects model if the *I*^2^ statistic was > 25%, and a fixed model was used if the statistic was < 25%. Pooling of data for clinical outcomes, stability measures, and functional testing was only performed if a minimum of three studies were available. The prevalence of osteoarthritis between groups was pooled as a binary yes/no variable and analyzed by calculating the odds ratios. If standard deviations were not reported, the standard deviation was calculated using the following formula: SD = max–min/4 [10, 18]. All tests of significance were two-tailed, and an *α* of less than 0.05 was considered significant. Publication bias was assessed using funnel plots and Egger’s test. Funnel and forest plots, and all statistical analyses, were performed using STATA SE (Version 13.0; StataCorp, College Station, Texas, USA) for Windows and the comprehensive meta-analysis software package (CMA), version 3 (Biostat Inc., Englewood, NJ, USA).

## Results

### Study selection and characteristics

**Fig. 1:**
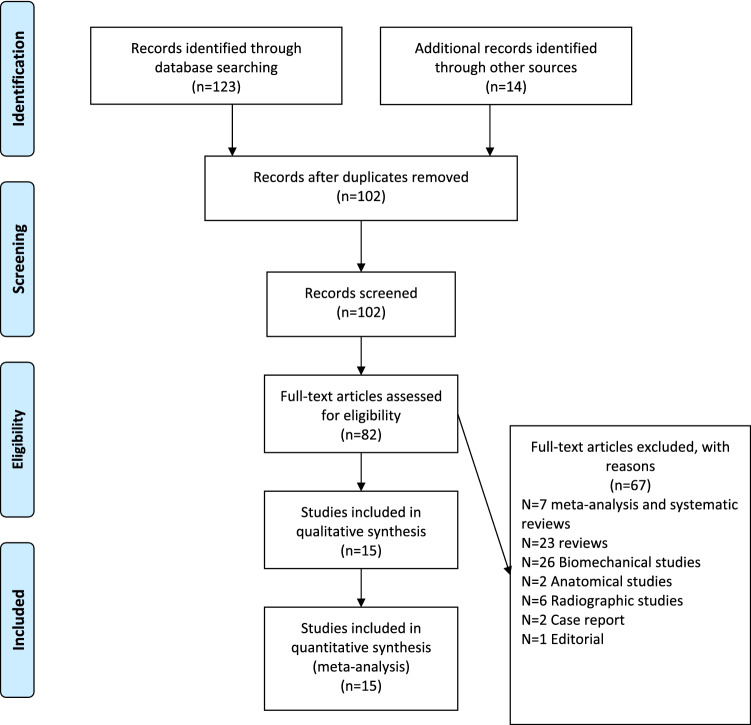
PRISMA flow diagram. From the initial 137 records, 15 studies were included in the quantitative synthesis

The initial literature search identified 137 studies for consideration. Of those, 35 studies were excluded for duplication, and the titles of the remaining 102 publications were checked for eligibility. Another 20 studies were excluded, and the full-text manuscripts of 82 studies were examined. Fifteen studies met all of the eligibility criteria and were included in the analysis (Fig. 1) [19–33]. Four studies were level II evidence [19–22], and the remaining eleven studies were level III evidence [23–33]. All 15 studies were published in English between 2013 and 2022, with a cumulative total of 682 cases. A total of 360 cases were treated with a hook plate, and 322 cases were treated with double-button fixation. The study characteristics are summarized in Table 1. Overall agreement between the two reviewers for final eligibility was excellent (kappa value 0.94, 95% CI 0.91–0.97).

**Table 1:** Summary of all included studies

Authors	LOE	Country	Patients (*n*) Hook-button	Age (years) Surg-Cons	Gender	Rockwood classification	Follow-up (months)	Outcome Hook-button
Sokkar [19]	II	Egypt	10 10	35.2 + 8.2	M = 15 F = 5	III	11.7 + 1.6 11.3 + 2.6	UCLA 32.2 + 2.33–32.5 + 2.22 VAS 9.2 + 1.03–9.3 + 1.0
Cai [20]	II	China	39 30	41.8 + 10.2 42.8 + 11.9	M = 26 F = 13 M = 19 F = 11	III	12	Constant 92.6 + 6.4–92.0 + 6.7 VAS 1.92 + 1.11–0.97 + 1.03
Pongsamakthai and Tharakulphan [21]	II	Thailand	22 22	39.6 + 9.6 37.1 + 11.5	M = 17 F = 5 M = 16 F = 6	III–V	3	Constant 81.6 + 5.7–90.4 + 3.5
Stein [22]	II	Germany	27 39	37.6 + 9.7 34.2 + 9.7	M = 26 F = 1 M = 28 F = 1	III–V	40.1 30.7	Taft 9.41 + 1.69–10.9 + 1.08 Constant 90.2 + 7.8–95.3 + 4.4 VAS 2.44 + 2.99–1.74 + 1.86
Andreani [23]	III	Italy	9 19	32.3 (19–60)	M = 22 F = 8	IV-VI	24 (6–48)	Constant 75 (65–85)–90 (74–99) UCLA 80–95
Metzlaff [24]	III	Germany	20 24	37.6 (18–56)	M = 44 F = 9	III–V	32 (24–51)	Constant 92.8 + 3.8–93.6 + 3.4 Taft 10.5 + 1.2–10.9 + 0.9
Jensen [25]	III	Germany	30 26	39 (18–68) 39 (18–54)	M = 28 F = 2 M = 23 F = 3	III	48 (7–77) 17 (7–29)	Constant 88 (20–99)–89 (52–100) Taft 10 (3–12)–10 (5–12) VAS 1.7 + 2.3–1.3 + 1.8 SST 10.4 + 2.8–11.0 + 1.3
Natero-Cisneros [26]	III	Spain	11 20	41 (19–55) 36 (25–52)	M = 11 F = 0 M = 17 F = 3	III–V	32.5 + 11.6 38.4 + 4.3	VAS 1.45 + 1.51 Constant 91.4 + 6.84–95.3 + 0.93 DASH 4.8 + 5.6–3.0 + 2.0
Razak [27]	III	Singapore	10 16	49.2 + 16.9 41.4 + 12.3	M = 6 F = 4 M = 9 F = 1	III–V	23 (14–35)	Constant 48.1 + 26.9–38.7 + 18.2 OSS 14.3 + 3–13.3 + 5 VAS 7.3 + 7.9–3.3 + 5.5
Taleb [28]	III	Iran	8 9	39.6 + 16.2 37.3 + 12.02	M = 7 F = 1 M = 8 F = 1	III–IV	19.8 + 8.8 23.8 + 19.2	VAS 7.6 + 9.5–3.2 + 8.9 DASH 73.1 + 22.1–39.4 + 11 ASES 47.9 + 27.4–87.6 + 10.4 UCLA 21 + 7–32.2 + 1.4 Constant 55 + 22.9–91.7 + 5.9 SST 5.3 + 3.2–10.5 + 1.45
Li 2020	III	China	84 28	36.0 + 8.3 35.9 + 7.9	M = 63 F = 21 M = 21 F = 7	III–V	24	VAS 1.8 + 1.1–1.2 + 0.6 Constant 83.3 + 8.8–89.3 + 4.2
Fosser [30]	III	Italy	22 22	48.2 (22–70) 40.5 (21–63)	M = 16 F = 6 M = 20 F = 2	III–V	41.3 (6–58) 32.2 (6–46)	Constant 92.7 (80–100)–96.1 (72–100)
Shen [31]	III	China	19 16	40.2 + 8.7 44.9 + 11	M = 10 F = 9 M = 10 F = 6	III, IV	30 (16–40) 27 (15–42)	Constant 93.7 + 6.6–95.7 + 7.3 VAS 0.7 + 0.6–0.4 + 0.6 UCLA 33.7 + 1.5–33.9 + 2.5
Yoo [32]	III	Korea	10 12	44.4 + 6.5 42.8 + 5.5	M = 7 F = 3 M = 8 F = 4	III, IV	31.5 (24–62)	VAS 1.14 + 1.45–1.02 + 1.32 ASES 72.2 + 12.96–86.32 + 13.46 SPADI 35.2 + 11.5–23.2 + 10.5 DASH 38.8 + 7.6–22.2 + 12.5
Liu [33]	III	China	39 32	41.8 + 10.5 39.6 + 8.9	M = 29 F = 10 M = 23 F = 9	III	12	Constant 94.4 + 3.2–94.8 + 3.5 DASH 4.5 + 3.3–4.6 + 3.9 VAS 0.4 + 0.8–0.3 + 0.8

### Risk of bias and quality assessment

The findings of the risk of bias assessment are summarized in Tables 2 and 3.

**Table 2:**
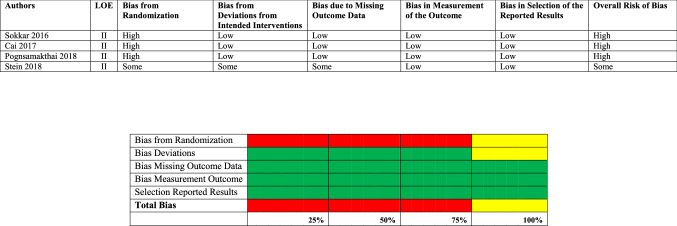
Risk of bias Cochrane risk of bias assessment tool version 2

**Table 3:** Risk of bias ROBINS Cochrane risk of bias assessment tool version 2

Authors	LOE	Bias due to confounding	Bias in selection of participants	Bias in classification of interventions	Bias due to deviations from intended interventions	Bias due to missing data	Bias in measurement of outcomes	Bias in selection of the reported results	Overall bias
Andrani 2013	III	Moderate	Moderate	Serious	Moderate	Low	Low	Low	Serious
Metzlaff [24]	III	Moderate	Moderate	Moderate	Low	Low	Low	Low	Moderate
Jensen [25]	III	Moderate	Low	Low	Low	Low	Moderate	Low	Moderate
Natero-Cisneros [26]	III	Moderate	Serious	Moderate	Low	Moderate	Low	Low	Serious
Razak [27]	III	Moderate	Low	Low	Low	Low	Low	Low	Moderate
Taleb [28]	III	Critical	Critical	Low	Low	Low	Low	Low	Critical
Li 2020	III	Moderate	Moderate	Low	Low	Low	Low	Low	Moderate
Fosser [30]	III	Moderate	Moderate	Serious	Low	Low	Low	Low	Serious
Shen [31]	III	Moderate	Moderate	Low	Low	Low	Low	Low	Moderate
Yoo [32]	III	Moderate	Moderate	Serious	Low	Serious	Low	Low	Serious
Liu [33]	III	Moderate	Moderate	Low	Low	Low	Low	Low	Moderate

#### Risk of bias Cochrane assessment tool version 2

Three of the LOE II studies [19–21] were assessed as having a high risk of bias. The high risk was caused by bias from randomization. Sokkar et al. [19] have described in their methods section that they randomly divided the patient cohort, but failed to provide any detailed information. Cai et al. [20] have apparently performed a prospective randomized trial, but failed to provide any specific information in their manuscript. Pongsamakthai et al. [21] used a sealed envelope and box size techniques, but also failed to provide any detailed information regarding how randomization was executed. Stein et al. [22] used a web-based randomizer but also failed to provide further details, and their randomization protocol was, therefore, assessed as having some risk.

#### Risk of bias ROBINS-I assessment tool

For the eleven observational studies, the ROBINS-I tool was used to assess the risk of bias [23–33]. The study by Taleb et al. [28] was assessed as having a critical risk of bias, after they performed a retrospective study where group allocation was unclear. This assessment has resulted in an overall critical risk of bias. Four studies [23, 26, 30, 32] were assessed as having a serious risk of bias, with bias in the classification of interventions [23, 26, 30]; the intervention groups were not clearly defined in these manuscripts. Two studies [30, 32] were assessed as having serious risk due to missing data. Fosser et al. [30] included 120 patients initially, but only 31 patients were considered in the final analysis. Yoo et al. [32] included 38 patients initially, but only 22 were considered in the final analysis.

**Fig. 2:**
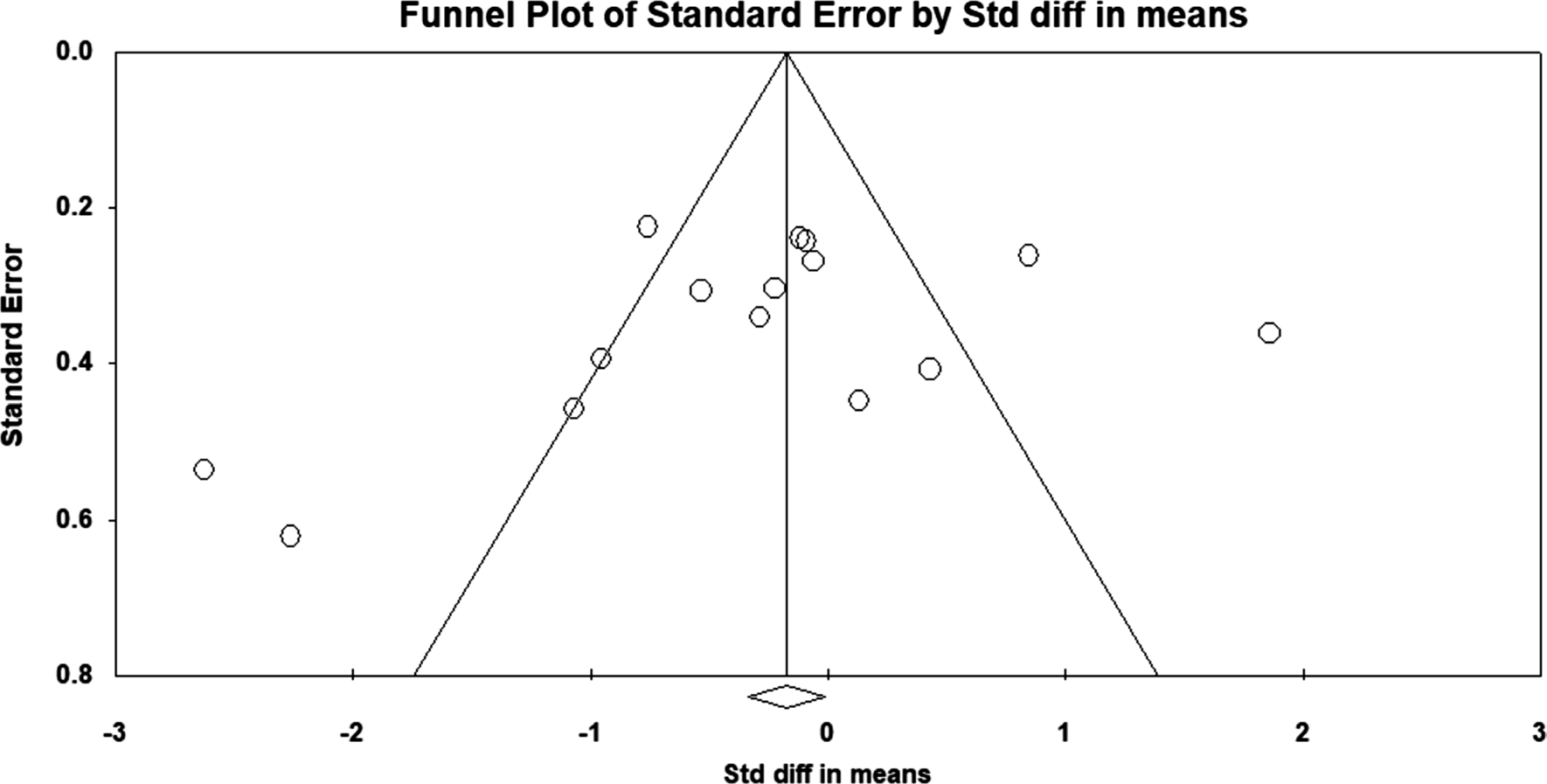
Publication bias: Publication bias was not detected. The funnel plot was symmetric, and Egger’s regression intercept (intercept − 2.91, *t*-value 1.18, *p*-level 0.257) was not significant

Publication bias was not detected. The funnel plot was symmetric, and Egger’s regression intercept (intercept − 2.91, *t*-value 1.18, *p*-level 0.257) was not significant (Fig. 2).

#### Quality assessment

The GRADE quality assessment for all included outcomes is summarized in Table 4. All outcomes were downgraded to very low quality, related to inconsistency of results due to a considerable *I*^2^, high risk of bias, and missing 95% confidence intervals.

**Table 4:** Quality assessment using the Cochrane GRADE system

Authors	Initial level of certainty	Final level of certainty	Risk of bias	Inconsistency of results	Indirectness of evidence	Imprecision of results	Large effects (upgrading)	Dose response (upgrading)	Opposing plausible residual bias and confounding (upgrading)
Clinical outcomes all studies	Low	Very low	High	Considerable* I*^2^	N/A	95% CI missing	N/A	N/A	N/A
Clinical outcomes LOE II studies	High	Very low	High	Considerable* I*^2^	N/A	95% CI missing	N/A	N/A	N/A
Clinical outcomes LOE III studies	Low	Very low	High	Considerable* I*^2^	N/A	95% CI missing	N/A	N/A	N/A
VAS all studies	Low	Very low	High	Considerable* I*^2^	N/A				
VAS LOE II studies	Low	Very low	High	Considerable* I*^2^	N/A	95% CI missing	N/A	N/A	N/A
VAS LOE III studies	Low	Very low	High	Considerable* I*^2^	N/A	95% CI missing	N/A	N/A	N/A

The MINORS score (Table 5) was above 16 indicating good study quality for three LOE II studies [20, 22] and for two LOE III studies [24–27]. Fair study quality was observed in eight LOE III studies [25, 26, 28–33]. One LOE II study [19] and one LOE III study [23] were considered low quality. The modified Coleman Score (Table 6) demonstrated good study quality for one study [25], fair study quality for eight studies [20–22, 24, 26, 29, 30, 33], and poor quality for six studies [19, 23, 27, 28, 31, 32].

**Table 5:** MINORS quality assessment

Authors	Total points	Clearly stated aim	Inclusion of consecutive patients	Prospective collection of data	Appropriate endpoints	Unbiased assessment	Follow-up appropriate	Loss of follow-up < 5%	Sample size calculation	Control group	Contemporary groups	Baseline group equivalence	Adequate statistical analysis
Sokkar [19]	7	0	2	0	2	0	1	0	0	0	2	0	0
Cai [20]	17	2	2	2	2	0	1	2	0	0	2	2	2
Pongsamakthai and Tharakulphan [21]	19	2	2	2	2	0	1	2	2	0	2	2	2
Stein [22]	18	2	2	2	2	0	2	0	2	0	2	2	2
Andreani [23]	8	0	2	0	2	0	2	0	0	0	2	0	0
Metzlaff [24]	16	2	2	0	2	0	2	2	2	0	2	0	2
Jensen[25]	11	2	2	0	2	0	1	0	0	0	2	0	2
Natero-Cisneros [26]	10	2	0	0	2	0	2	0	0	0	2	0	2
Razak [27]	18	2	2	2	2	0	0	2	2	0	2	2	2
Taleb [28]	11	2	0	0	2	0	1	0	0	0	2	2	2
Li 2020	12	2	0	0	2	0	2	0	0	0	2	2	2
Fosser [30]	12	2	0	0	2	0	2	0	0	0	2	2	2
Shen [31]	13	2	0	0	2	0	2	1	0	0	2	2	2
Yoo [32]	12	2	0	0	2	0	2	0	0	0	2	2	2
Liu [33]	10	0	0	0	2	0	1	1	0	0	2	2	2

**Table 6:** Modified Coleman Methodology Score

Authors	Total points	Study size	Mean follow-up	Percent of patients with follow-up	Number of interventions	Type of study	Diagnostic certainty	Description surgical technique	Description postoperative rehabilitation	Outcome criteria	Procedures for assessing outcomes	Description of subject selection
Sokkar [19]	43	0	0	3	10	10	5	5	0	10	5	0
Cai [20]	64	4	0	5	10	15	5	5	0	10	5	5
Pongsamakthai and Tharakulphan [21]	64	4	0	5	10	15	5	5	0	10	5	5
Stein [22]	69	4	0	5	10	15	5	5	0	10	5	10
Andreani [23]	45	0	0	5	10	0	5	5	0	10	5	5
Metzlaff [24]	60	0	5	10	10	0	5	5	5	10	5	5
Jensen [25]	73	4	5	10	10	0	5	5	5	10	9	10
Natero-Cisneros [26]	60	0	0	5	10	0	5	5	5	10	5	15
Razak [27]	55	0	0	5	10	10	5	5	5	10	5	5
Taleb [28]	43	0	0	3	10	5	5	0	0	10	5	5
Li 2020	60	7	0	3	10	0	5	5	5	10	5	10
Fosser [30]	59	4	0	5	10	0	5	5	5	10	5	10
Shen [31]	54	0	0	5	10	0	5	5	5	10	9	5
Yoo [32]	45	0	0	0	10	0	5	5	5	10	5	5
Liu [33]	64	4	0	5	10	5	5	5	10	10	5	5

### Clinical outcomes

**Fig. 3:**
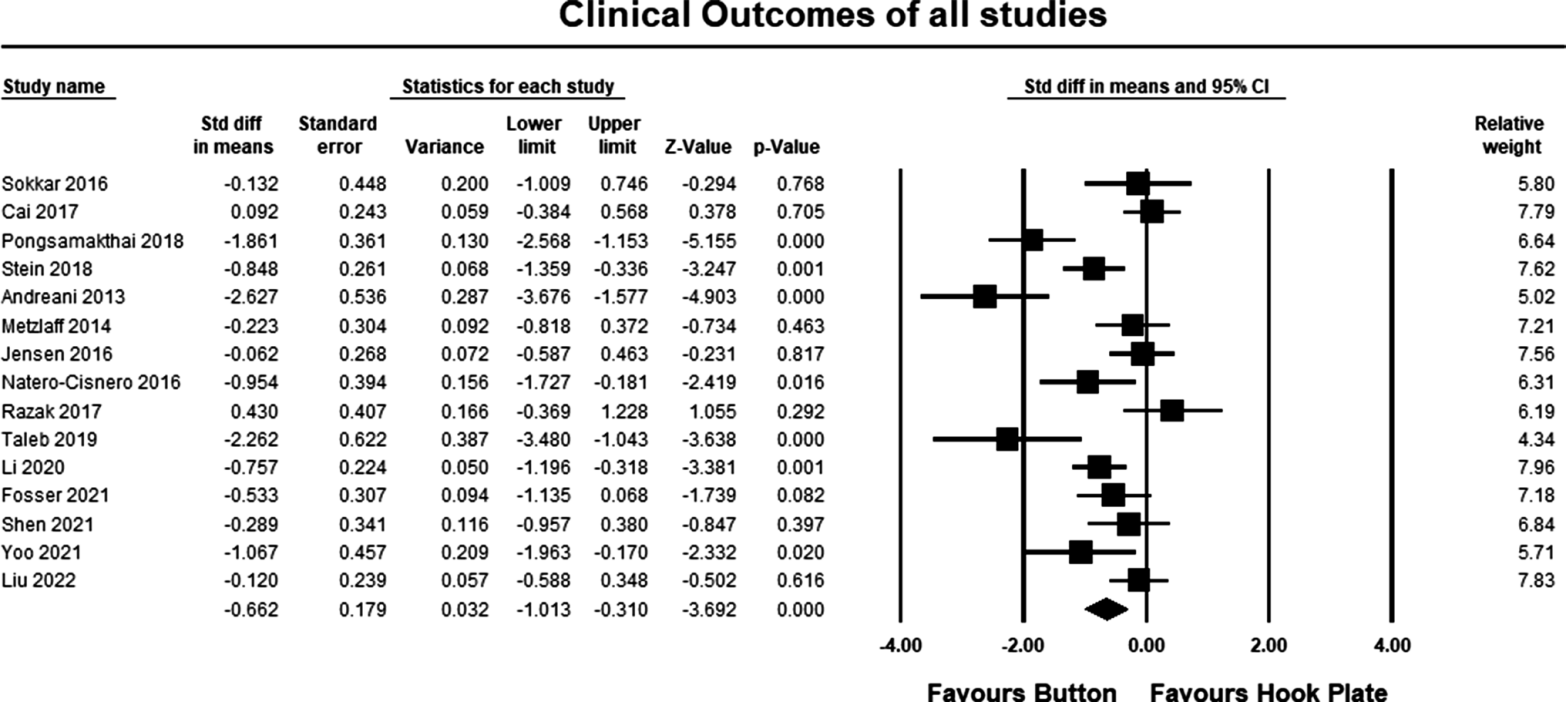
Forest plot comparing clinical outcomes for all studies. The pooled estimate for all studies was statistically significant (*p* = 0.0001) in favor of button repair

**Fig. 4:**
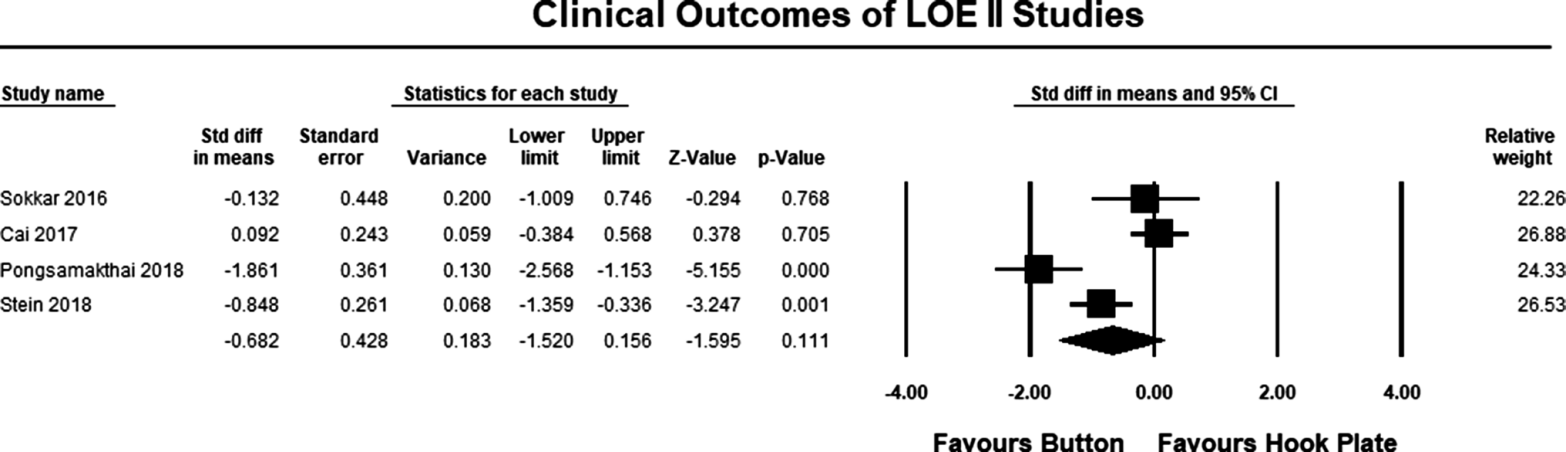
Forest plot comparing clinical outcomes for LOE II studies. The pooled estimate for all studies could not statistically significant (*p* = 0.11)

**Fig. 5:**
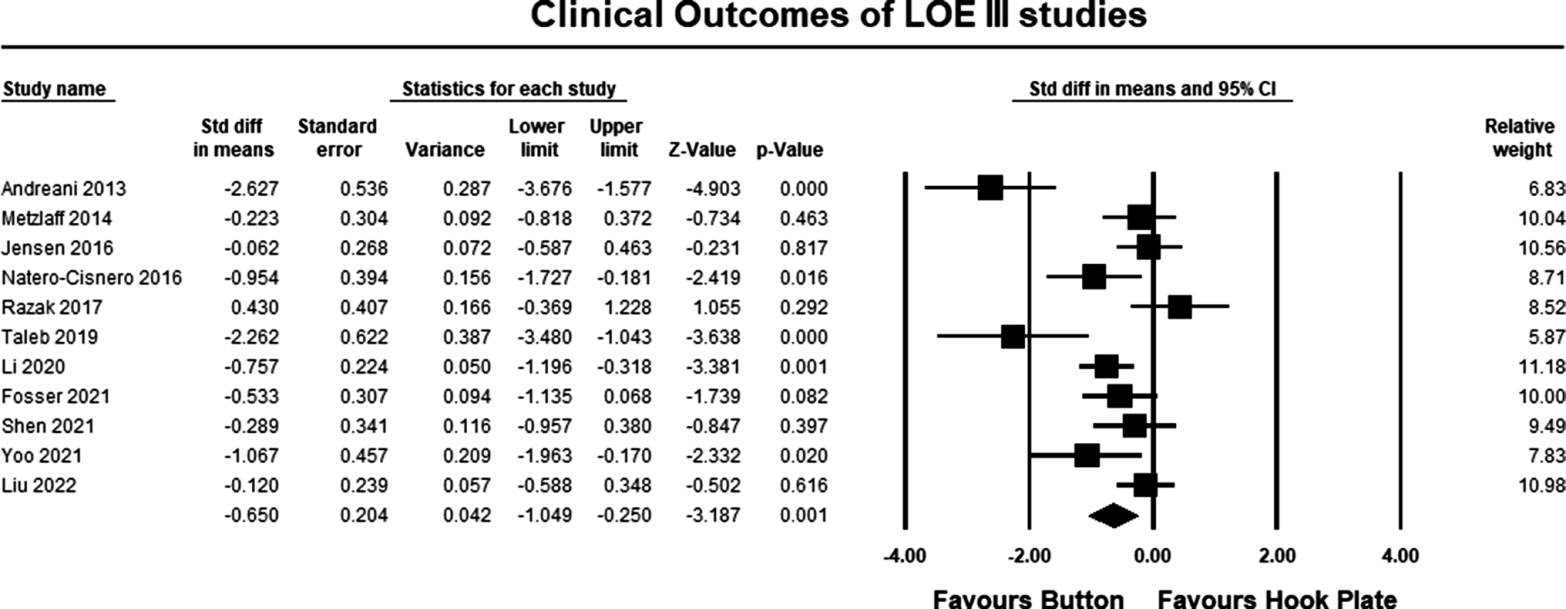
Forest plot comparing clinical outcomes for LOE III. The pooled estimate for all studies was statistically significant (*p* = 0.001) in favor of button repair

The clinical outcomes for all studies are summarized in Table 1. The pooled estimate for both LOE II and LOE III studies demonstrated significant differences between the hook plate and double-button fixation, in favor of double-button fixation (SMD − 0.662, 95% CI − 1.013 to − 3.692, *p* = 0.0001, *I*^2^ = 74%; Fig. 3). According to Cohen, the magnitude effect is medium, suggesting that more than 69% of the results with the hook plate are below the results when using double-button fixation [34]. The pooled estimate for LOE II studies was similar to the pooled results for LOE II and III studies but was not statistically significant (SMD − 0.682 95% CI 0.183 to − 1.595, *p* = 0.111, *I*^2^ = 96%; Fig. 4). According to Cohen, the magnitude effect is medium, suggesting that more than 69% of the results with the hook plate are inferior to the results when using double-button fixation [34]. The failure to reach significance can possibly be explained by study heterogeneity and the lack of a statistically significant between group differences in two studies [19, 20]. The pooled estimate for LOE III studies was similar to the pooled results for LOE II and III studies and was statistically significant in favor of double-button fixation (SMD − 0.650, 95% CI − 1.049 to − 3.187, *p* = 0.001, *I*^2^ = 56%; Fig. 5). According to Cohen, the magnitude effect is medium, suggesting that more than 69% of the results with the hook plate are inferior to the results when using double-button fixation [34].

### Clinical outcomes for pain

**Fig. 6:**
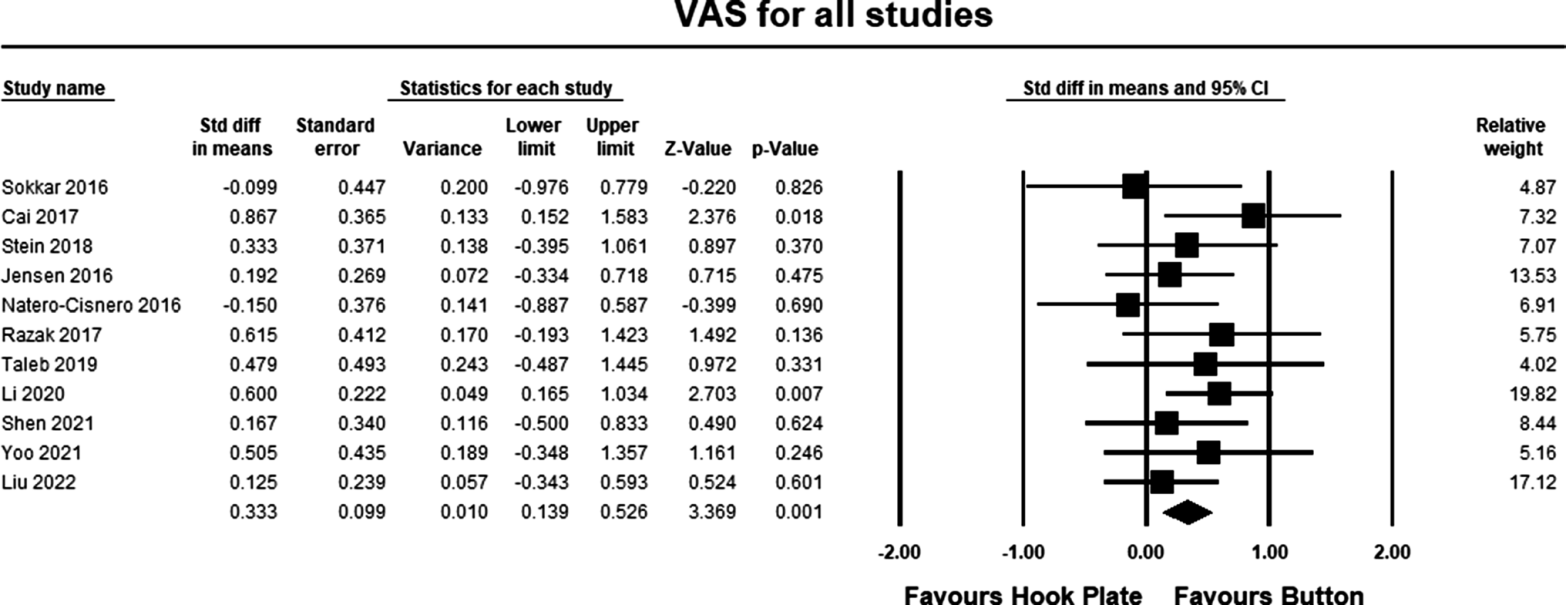
Forest plot comparing outcomes for pain for all studies. The pooled estimate for all studies was statistically significant (*p* = 0.001) in favor of button repair

Eleven studies [19, 20, 22, 25–29, 31–33] included pain assessment scales in their study. The pooled estimate for both LOE II and LOE III studies demonstrated significant differences between the hook plate and double-button fixation in favor of double-button fixation (SMD 0.333, 95% CI 0.139–3.369, *p* = 0.001, *I*^2^ = 85%; Fig. 6). According to Cohen, the magnitude effect is between medium and small, suggesting that between 58 and 69% of the results with the hook plate were inferior to the results when using double-button fixation [34].

**Fig. 7:**
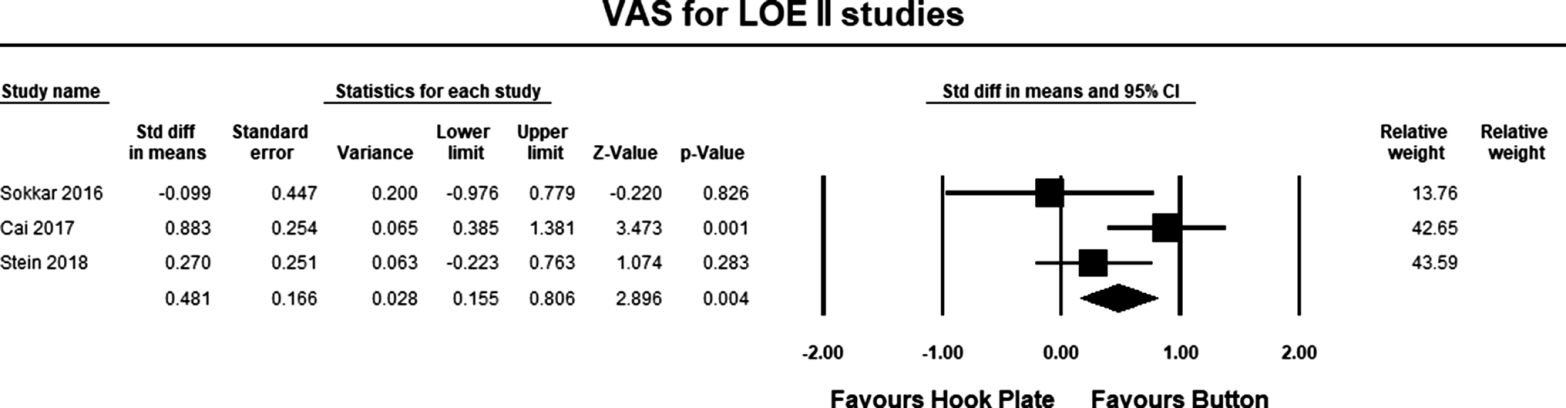
Forest plot comparing outcomes for pain for LOE II studies. The pooled estimate for all studies was statistically significant (*p* = 0.004) in favor of button repair

Three LOE II studies [19, 20, 22] included pain assessment scales in their study. The pooled estimate for LOE II studies demonstrated significant differences between the hook plate and double-button fixation in favor of double-button fixation (SMD 0.481, 95% CI 0.155–0.806, *p* = 0.004, *I*^2^ = 59%; Fig. 7). According to Cohen, the magnitude effect is small, suggesting that only 58% of the results with the hook plate were inferior to the results when using double-button fixation [34].

**Fig. 8:**
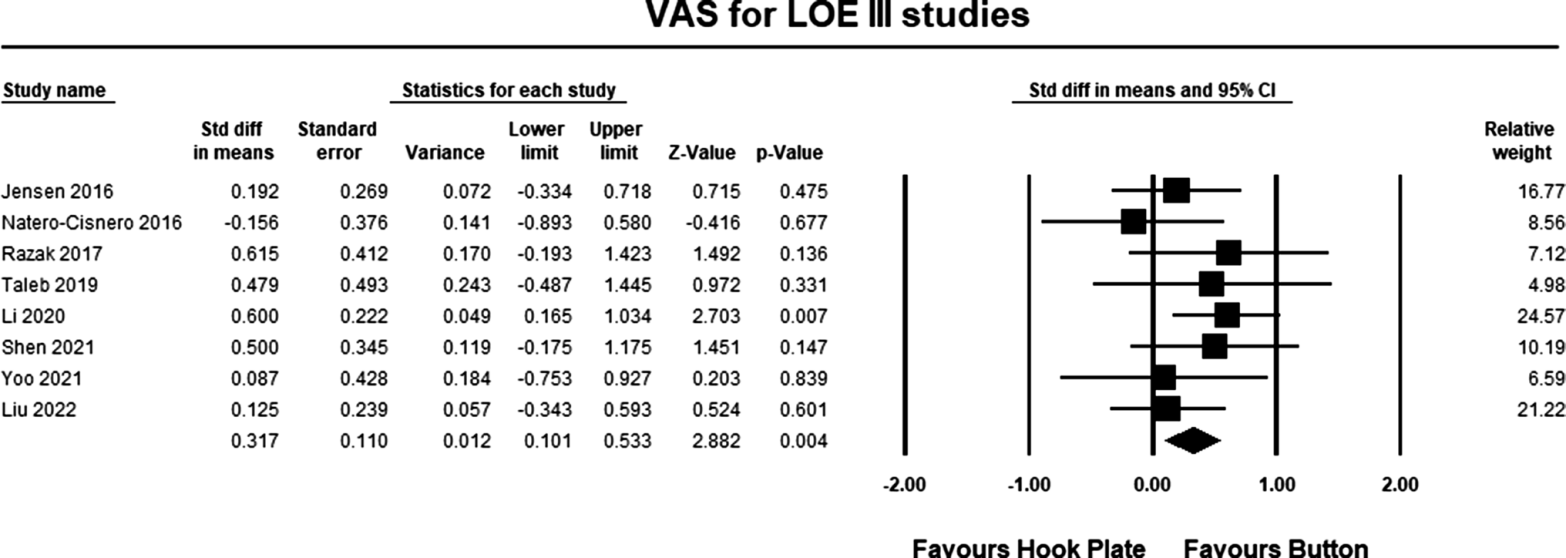
Forest plot comparing outcomes for pain for LOE III studies. The pooled estimate for all studies was statistically significant (*p* = 0.004) in favor of button repair

Eight LOE III studies [25–29, 31–33] included pain assessment scales in their study. The pooled estimate for LOE III studies demonstrated significant differences between the hook plate and double-button fixation in favor of double-button fixation (SMD 0.317, 95% CI 0.012–2.882, *p* = 0.004, *I*^2^ = 0%; Fig. 8). According to Cohen, the magnitude effect is between small and medium, suggesting that between 58 and 69% of the results with the hook plate were inferior to the results of when using double-button fixation [34].

### Complications

**Fig. 9:**
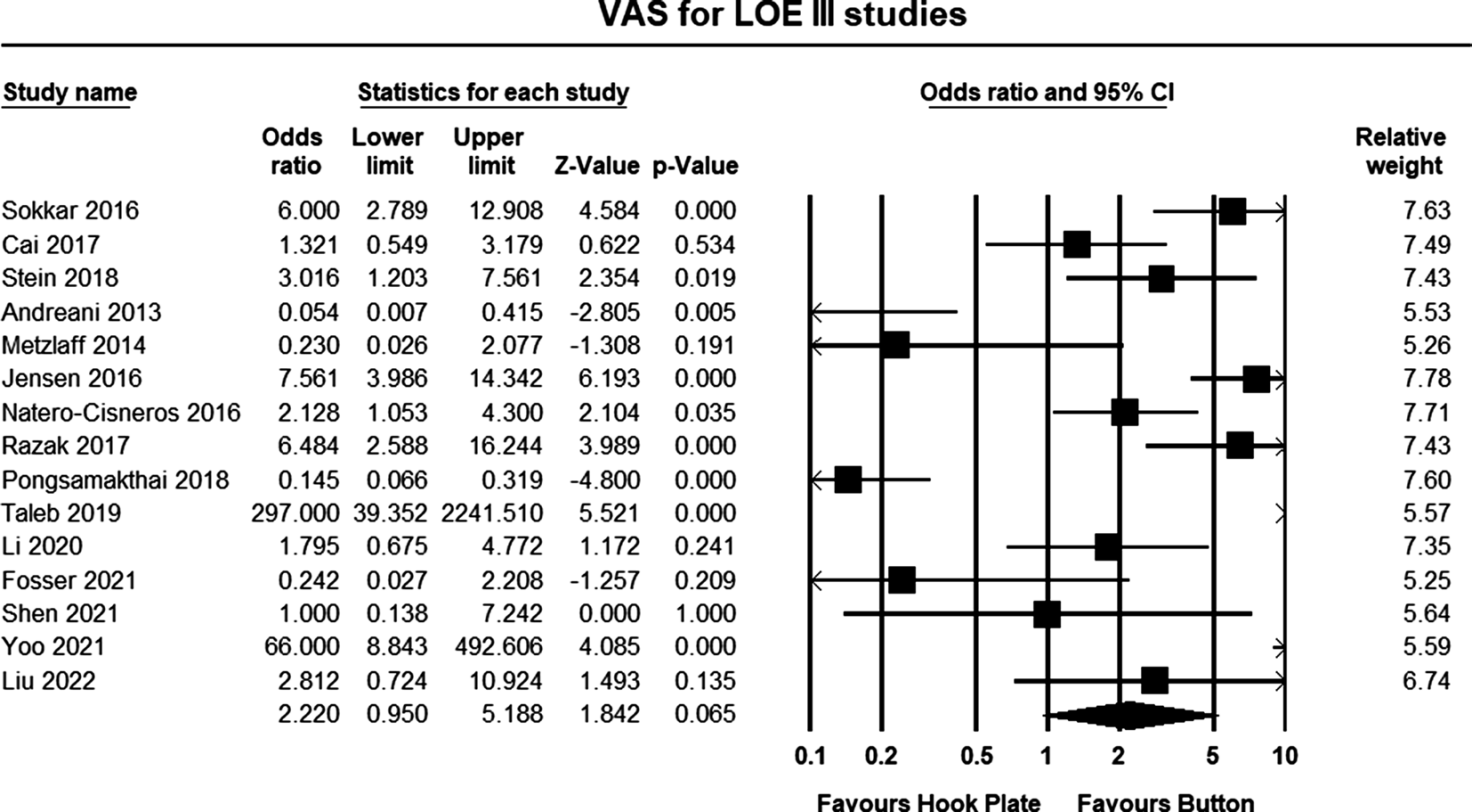
Forest plot comparing complications for all III studies. The pooled estimate for all studies was not statistically significant (*p* = 0.065) but favored button repair. The odd ratio indicated that complications were twice as likely in the clavicle hook plate

All studies reported complication rates (Table 7). The pooled estimates demonstrated overall lower complication rates in the double-button group, but the between-group differences did not reach statistical significance (odds ratio 2.220, 95% confidence intervals 0.95–5.188, *p* = 0.065) (Fig. 9).

**Table 7:** Complications

Authors	Hook plate	Button	Percentage hook plate (%)	Percentage button (%)
Sokkar [19]	Infection 2 Osteolysis 2	Failure 1	40	10
Cai [20]	Infection 3 Nerve injury 1 Hardware related 1	Re-dislocation 0–3	12.8	10
Stein [22]	Horizontal instability: 5	Horizontal instability: 3	18.5	7
Andreani [23]	None reported	Pain 1 Hardware related 2	0	15.8
Metzlaff [24]	None reported	Loss of reduction 1	0	4.2
Jensen [25]	Wound complications 12 ACJ OA 8 Hardware related 4 Erosion 6	ACJ OA 5 Loss of reduction: 3 Hardware related 1	80	34.6
Natero-Cisneros [26]	Erosion 1 Implant failure 1 Infection 1	Implant failure 1 Infection 2	27.3	15
Razak [27]	Plate cut-out 1 Erosion 2	Infection 2	30	6.2
Pongsamakthai and Tharakulphan [21]	Late 1 Loss of reduction 1	Early 3 Late 1 Loss of reduction 5	9.1	40.9
Taleb [28]	Erosions 6	None reported	75	0
Li 2020	Infection 2 Loss of reduction 10	Loss of reduction 2	11.9	7.1
Fosser [30]	None reported	Recurrence 1	0	4.5
Shen [31]	Acromial erosion: 1	Loss of reduction 1	2.1	2.0
Yoo [32]	ACJ OA 4	None reported	40	0
Liu [33]	Shoulder pain 1 Osteolysis 1 Distal clavicle fracture 1	Infection 0–1	7.7	3.1

## Discussion

The results of this meta-analysis clearly demonstrated superiority of the double-button repair compared to the clavicle hook plate. For clinical outcomes, the pooled estimates for all studies demonstrated that more than 69% of all double-button repairs had better clinical outcomes when compared to the hook plate. This result was also observed when separately pooling LOE II and LOE III studies. Similarly, patients who were treated with double-button repair had significantly less pain, but the treatment effect was lower and 58–69% of the double-button group had substantially less pain than patients treated with the hook plate. Double-button repair was also associated with a 2.2 times lower overall risk for complications.

An earlier meta-analysis included four studies and concluded that both techniques resulted in good clinical outcomes, but reported that double-button fixation had an advantage with regard to postoperative pain [9]. Compared to Pan et al. [9], our meta-analysis has included a total of 15 studies, increasing the sample size by 400%. Pan et al. [9] have included two case–control, one cohort, and one randomized controlled trial, and pooled all studies. It could be argued that the inclusion of observational studies is typically associated with high risk of bias, heterogeneity, and could be unbalanced on confounding factors. Therefore, non-randomized studies should theoretically not be included into meta-analysis. The Cochrane Handbook outlines that high risk of bias for one or more key domains is sufficient to weaken the confidence in the interpretation of the results with pooling randomized controlled trials [10]. However, Anglemyer et al. [35] compared outcomes between observational and randomized trials. In their review, they have demonstrated that there is little evidence that there would be significant effect estimate differences between observational studies and RCTs, regardless of study design and heterogeneity [35]. Abraham et al. reported that the mean difference between RCTs and non-randomized comparative studies was between 4.0 and 5.6%, and concluded that the inclusion of non-randomized studies into meta-analysis is possibly as accurate as that of RCTs alone [36]. Bun et al. investigated the effect of pooling different study types and concluded that treatment effects did not differ between observational studies and RCTs as long as the same outcomes are pooled [14]. In this meta-analysis, studies were analyzed according to the level of evidence, but also pooled into one cumulative analysis. The effect estimate did not differ between the three analyses, indicating that the true treatment effect was not influenced by the inclusion of observational studies. However, only with the analysis of LOE II studies did the findings not reach statistical significance. This could be explained by a small sample size and bias by sampling error, resulting in low power and type II error [37]. The inclusion of observational comparative studies has increased the overall sample size reducing the risk of type II error, and as a consequence resulted in significant between-group differences in favor of double-button repair. The increase in sample size has also increased the external validity and generalizability of the findings.

Unfortunately, the risk of bias within and across studies was assessed as high, and the Cochrane Handbook specifies that high risk of bias can affect the interpretation of results [10]. In this meta-analysis, the risk of bias was high for 75% of the LOE II studies and serious or critical for 45% of the included observational studies. These conclusions should, therefore, be interpreted with caution. Study quality was assessed using the MINORS scoring system and modified Coleman Methodology Score for within study quality, and the GRADE system for across studies outcome variables. The study quality with GRADE was assessed as very low for each outcome across studies. Considerable heterogeneity and imprecision of results by omitting the 95% confidence intervals were the main reasons. According to Cochrane, this reduces the confidence in the effect estimate [10]. In addition, further high-quality studies are likely to have an impact on the estimate of the treatment effect and may actually change the estimate. Within study quality using two validated instruments revealed that the majority of studies were of fair and poor, quality further reducing confidence and validity.

Despite a large number of studies describing numerous techniques to treat ACJ injuries, a widely accepted standard does not exist. Certainly, there has been a focus on more precise restoration of normal anatomy using minimally invasive techniques, taking both horizontal and vertical instability into consideration [38]. More temporary techniques for acute injuries include arthroscopic-assisted or minimally invasive coracoclavicular reconstructions with button suture configurations, combined with rigid fixation with clavicle hook plates [2, 39].

Clinical outcomes are significantly better in the double-button repair group, and despite the concerns with risk of bias, study quality, and heterogeneity, one could argue that there is a strong argument in favor of suture-button repair when surgical repair is indicated for acute acromioclavicular joint injuries. Superiority of double-button fixation is also observed with postoperative pain, and patients in the double-button group had significantly lower pain scores, although the treatment effect was lower. The possible explanation for this finding could be less soft-tissue disruption during surgery with double-button fixation in the early postoperative stage. Significantly lower pain levels were also observed more than 12 months postoperative for double-button repair, and the reasons for these differences are not clear. Complication rates are an important factor when deciding which of the two surgical techniques should be considered. Clearly, the complication rates are significantly lower for double-button repair, and in the hook plate group, complications were twice as high compared to the button repair group. Loss of reduction, recurrence, and horizontal instability were the main complications in the button group, while wound complications, infection, and osteolysis/acromial erosions were the main complications in the hook plate group. Another potential disadvantage of the hook plate is the need for plate removal.

Despite the recent dramatic increase in the number of publications on acromioclavicular injuries, controversy still exists with regard to diagnosis, classification, and optimal treatment [38, 39]. Given the lack of high-level evidence, the decision to operate or not and which technique to use is ultimately a joint decision between patient and surgeon, and must be based on individual patient characteristics, professional activities and demands, their desire to return to sports, and their willingness to adhere to the postoperative rehabilitation protocol [2].

### Limitations

This meta-analysis has several limitations. The included studies had small to moderately small sample sizes with variable follow-up intervals, introducing sampling bias and heterogeneity. Subgroup analysis considering the severity of dislocation (Rockwood III–VI) was not performed, as this would have resulted in low power. Another limitation is that this meta-analysis focused on two more contemporary techniques, and it is possible that this approach has caused selection bias. In addition, non-operative treatment was not included and may have been appropriate for Rockwood type III injuries with similar outcomes. It could be argued that the inclusion of both randomized controlled trials and comparative observational studies has resulted in unbalanced trial groups, with confounding factors introducing bias. Even though the search strategy included multiple databases, it is possible that studies were missed. This could also be because the search was limited to English and German publications only. Publication bias can, therefore, not entirely be excluded; however, funnel plot and Egger’s intercept do not suggest that publication bias was introduced.

## Conclusions

The results of this meta-analysis demonstrated significantly better outcomes with double-button repair for acute ACJ dislocations when compared to using a clavicle hook plate. Double-button repair is also associated with a 2.2 times lower risk of complications; however, risk of bias is high, and study quality within and between studies was low. These results must, therefore, be viewed with caution.

## Data Availability

Not applicable.
